# Managing conflict styles to accelerate leadership effectiveness

**DOI:** 10.1186/s12919-024-00313-1

**Published:** 2024-12-05

**Authors:** Greg Pennington

**Affiliations:** Managing Partner, Pennpoint Consulting Group, Tucker, GA USA

**Keywords:** Managing conflict, Conflict styles, Leadership styles, Academic leaders, Effective leadership, Persons of color

## Abstract

When describing leadership effectiveness as influencing and impacting the feelings, thoughts and behaviors of others, it can be seen as a critical skill in the overall effectiveness of leaders in general, including those in higher education. Understanding what leadership skills contribute to differentiating between average leaders and more effective leaders, provides insights into where transitions from individual academic roles to leadership ones can be accelerated. In this article we share thoughts and an approach to identifying the importance of conflict management as a key leadership skill to increasing overall leadership effectiveness. We describe a workshop facilitated as a component of the Accomplishing Career Transitions (ACT) Program of the American Society for Cell Biology (ASCB). The workshop, A Leadership Primer uses the Thomas-Kilmann Instrument (TKI), individual reflection, peer coaching and goal setting to provide insight into the origin and impact of individual conflict management styles. While there is evidence indicating that the use of a Collaborative style provides more opportunities for effective leadership, the participants in the ACT were like other academic leader samples that showed more use of a Compromising style. The workshop and follow up sessions provided coaching support to identify origins of conflict styles and options for increasing flexibility to apply a range of conflict styles.

## Background

The American Society for Cell Biology (ASCB) has developed programs that focus on the professional development of scientists from underrepresented backgrounds in STEM [1]. In addition to academic training these programs are intended to build leadership skills that are considered critical to the success of tenure track faculty member and their overall capability to influence and impact others. The Accomplishing Career Transitions Program (ACT) is one example of programs designed to accelerate that development.

The ACT program provided learning opportunities in specific content areas based on what ASCB considered key components of being successful in academia. Grant writing is an example of programs provided. The workshop on leadership was developed based on research that suggested where to focus. The design was also influenced by the goal of using an experiential approach to development. The motivation for focused development for this population included a recognition that the gap in the development of leadership skills may be more significant for those from underrepresented groups who had less access to mentoring resources than others. The design was driven by expectations to accelerate development by building on existing skills, and by focusing on dimensions of leadership that were key differentiators between more effective leadership and less effective leadership.

Managing Conflict is one of the skills included in a framework developed by ACT that identifies skills often needed to successfully transition into tenure positions. Managing Conflict is defined as “navigating the conflicts that arise in day-to-day interactions while working with a team of trainees and/or collaborators”. The list of key skills also included: “Negotiating”, and “Managing Collaborations” [1]. These skill areas were interrelated and influenced the decision to focus on “Managing Conflict”. This Leadership Primer workshop was designed to focus on that important area of “Managing Conflict.”

This article outlines the Leadership Primer workshop presented during the ACT summer conference in 2023. This conference was the final one for this cohort of the ACT program. The article provides insights into how the workshop was developed and its overall conceptual design. It also provides observations of the participants and proposed applications of what was learned.

## Main text

Building a foundation for recognizing, understanding, and managing approaches to conflict.

### Objectives

There were four objectives for the workshop. These included: (a) identifying “why” be a leader; (b) identifying specific strengths to leverage; (c) identifying specific development areas on which to focus; and (d) outlining an action plan to intentionally address accelerating leadership effectiveness.

### Definitions of leadership

The session began with an opportunity for participants to articulate why they wanted to be a leader. Participants were asked to consider examples of individuals they considered to be effective leaders. Starting with this focus, was intended to have participants recognize that their personal experiences with leaders shaped their definitions and their expectations of how they might demonstrate leadership [2].

It was important at this stage to define what was meant by leader and leadership. It was also valuable to connect definitions of leadership to experiences with other leaders and to frameworks and frames of reference. The use of metaphors to describe leadership has contributed to building a foundation of understanding different concepts and their application [3]. This is further enhanced when the metaphor builds upon an existing frame of knowledge.

In each discussion there was an appreciation of how being identified as under-represented categories in the areas of STEM might impact those definitions and applications. Given that the audience’s primary focus was understanding the biology of cells and how to research and apply that understanding to real problems, the question was posed as to what they knew from their understanding of cell biology that might inform their understanding of leadership. To accelerate understanding about leadership in two critical dimensions – managing conflict and adapting to change, participants were invited to consider how cell biology could serve as a metaphor to leadership, to managing conflict, and to adapting to change.

A basic book on cell biology [4] served as a reference point for framing responses to three questions. The questions were (a) “What do you already know from cell biology about effective leadership; (b) “What do you already know from cell biology about managing conflict”; and (c) “What do you already know from cell biology about adaptation and resistance to change”. The following points were raised in response to each question.

In response to “What do you already know from cell biology about effective leadership”, the following points were raised: Structure and Function, Communication and Coordination, Adaptability, Diversity and Inclusion, and Continuous improvement. A more specific example of how one of these characteristics of cells, Structure and Function, reflected aspects of leadership is in the same way cells make up basic building blocks of organisms, individuals constitute the basic building blocks of an organization. The organization of cells into tissues, organs, and organ systems, are comparable to the organization of individuals into teams, departments, and overall institutions. Being effective as a leader includes understanding the importance of organizing and coordinating individuals to achieve overall organization goals. Similar connections were apparent between the other points about cells and effective leadership.

In response to “What do you already know from cell biology about managing conflict, the following points were raised: Communication and Signaling, Homeostasis and Balance, Adaptation and Resilience, Collaboration and Cooperation, and Resolution and Healing. Just as Communication and Signaling in a cell are essential elements to coordinating activities and responding to changes in the environment, individuals and organizations benefit from open and clear communication between parties involved in a conflict to effectively manage it. Just as cells use signaling molecules to send information and coordinate responses, individuals in conflict use effective communication to indicate their perspectives, needs and concerns to reach agreement.

Thirdly, in response to “What do you already know from cell biology about adaptation and resistance to change”, the following points were raised: Adaptation through gene expression, Cellular differentiation as a metaphor for specialization, Cellular response, and Cellular homeostasis. An example of the discussion regarding cell biology as a metaphor for adaptation and resistance to change included recognition by participants that cells can adapt to changes in the environment by altering gene expression. Similarly, individuals and organizations can adapt to change by adjusting their behaviors, strategies, and goals. The discussion also included points about how cellular homeostasis and the efforts to maintain the status quo might represent both positive and negative resistance to change.

Making connections between leadership and cell biology was helpful to assure participants they had a foundation and framework for understanding leadership. It was also important to provide a specific research-based model for leadership in higher education. Kouzes and Posner’s [5] model for leadership in higher education was presented for this purpose. Based on interviews, their research outlined the following key behaviors for effective leadership, noting that they were similar across a variety to settings: Model the Way, Inspire a Shared Vision, Challenge the Process, Enable Others to Act, and Encourage the Heart.

### Managing conflict – a critical differentiator for effective leadership

ASCB’s list of critical skills and competencies for effective leadership [1] include managing conflict. Stanley and Algert [6] conducted research that suggested that managing conflict was critical and that leaders benefited from understanding their leadership styles and how to increase their effectiveness in managing conflict. The author’s experience in leadership development programs for leaders in land-grant institutions and STEM leaders in higher education [7] includes data that shows the prevalence of 360 feedback indicating the need for conflict management skills. This research, along with conflict styles data from academic leaders in nursing and journalism schools, further illustrated opportunities for academic leaders to broaden their skill sets and increase their flexibility in applying a range of conflict styles. The Thomas-Kilmann Conflict Styles Indicator has been the most frequently used measurement of conflict styles in each of these samples [6, 7]. DiSC Profiles [8], EQ-I Assessments [9], Five Behaviors of Cohesive Teams [10], and Hogan Assessments [11] are other assessment instruments used by the author that include feedback on managing conflict. Each of these instruments provides insights about conflict styles in the context of broad models of personality and leadership behavior. The TKI Assessment was used because of its focus on conflict styles specifically.

Similar to articulating a frame of reference for defining leadership effectiveness, it was important to invite participants to consider early messages they experienced about conflict. Though participants had taken the Thomas- Kilmann Indicator Assessment (TKI) they had not yet received their individual results. Before doing so they were asked to identify what they considered as their predominant approach to managing conflict. Using the TKI model, those choices were Competing, Collaborating, Compromising, Avoiding, and Accommodating. Participants were asked to make their self-assessments based on their interpretations of those five words.

Having self-identified their predominant style, participants were paired with another person and asked to consider an outside perspective on their conflict management styles by discussing the following: Share a snapshot of a conflict moment that involves you; Ask your partner their perspective of what conflict style you demonstrated; Discuss what you thought was your style, and what your assessment said was your style. They were also asked to consider what would be their secondary approach. Report outs to the full group included insights about how conflict styles were reflections of early messages regarding conflict, how important it was to use real examples to identify their conflict styles, and the value of another person adding their perspective on how you handled a conflict situation. When asked to share self-assessments about their predominant conflict management style, Collaborating was the predominant response with one person volunteering they were most often using a Competing style. Another person offered that they were most likely to use the Avoiding style. Participants comments included acknowledging how they interpreted the words used to identify styles and the importance of context in determining what style to use.

Participants were then provided an overview of the TKI describing its underlying dimensions and its intended use. The TKI [12] instrument is based on two dimensions, assertiveness and cooperativeness. The styles are usually presented in the order listed above because it reflects the relative position of assertiveness and cooperativeness. The five styles were presented with the following definitions and examples of how they are demonstrated:

- COMPETING reflects high assertiveness and low cooperativeness. You are trying to satisfy your own needs and concerns at the other person’s expense. It can be demonstrated by you assuming you can only win when the other person does not.
- COLLABORATING reflects assertive and cooperative. You are trying to find a win–win situation that satisfies both your concerns and those of the other person. It can be demonstrated by you making sure others are heard and asserting your own position.
- COMPROMISING reflects both assertiveness and cooperativeness. You work to find an acceptable agreement that partially meets your concerns and the other person’s.
- AVOIDING reflects being unassertive and uncooperative. You are likely to sidestep the conflict without trying to satisfy your concerns or those of the other person. It can be demonstrated by you ignoring the issue.
- ACCOMODATING reflects being unassertive and cooperative. You try to satisfy the concerns of the other person at the expense of your own. In can be demonstrated by you focusing more on sustaining the relationship than on getting your concerns addressed.

**Fig. 1:**
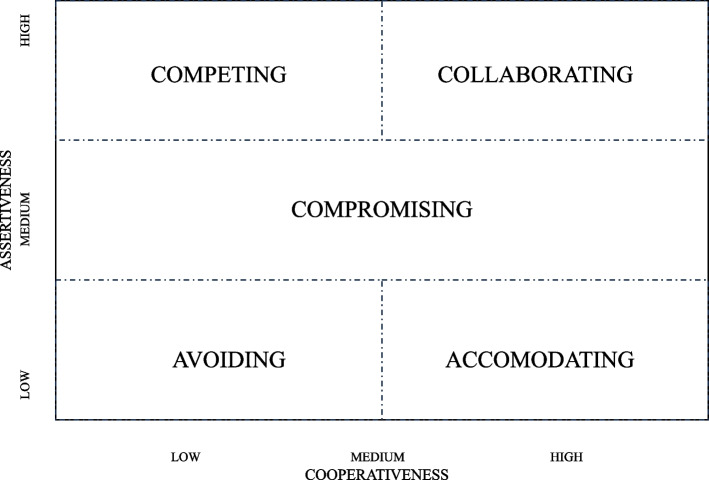
Thomas Kilmann conflict styles

Figure 1 shows the typical way the styles are presented.

As a measurement of how one responds to conflict situations, the TKI is intended to identify an individual’s preferred method of conflict resolution. One of the advantages of this model is increased self-awareness. This instrument allows individuals to have a greater understanding of how to approach conflict and emphasizes the value of having access to a range of styles in order to adjust to various situations.

## Results—conflict management styles

The distribution of conflict styles in the general population varies based on a number of factors and contexts. Insights from a number of leadership development programs including ones with academic leaders as participants suggest that 15–20% of the population may tend to demonstrate a Competing conflict style. Similar estimates suggest that 10–15% of the population may tend to demonstrate a Collaborating style and 20–25% exhibiting a preference for a Compromising style. Preferences for an Avoiding style are estimated to be demonstrated by 20–25% of the population and 10–15% may prefer an Accommodating conflict style.

Results for this cohort of participants in ACT showed the following distribution of preferred conflict styles. Included in Table 1 are the estimated ranges of percentage of preferred styles in the general population, actual percentages for a population of 22 leaders in journalism and mass communication, and actual percentages for a sample of 13 higher education leaders in STEM [7].

**Table 1:** TKI percentages of preferred conflict styles

TKI Style	General Population	Journalism and Mass Communication	ASCB	STEM
Competing	15–20	20	15	15
Collaborating	10–15	0	19	0
Compromising	20–25	30	35	38
Avoiding	20–25	10	19	15
Accommodating	10–15	40	11	32

Participants were invited to offer reflections and ask questions about the data. Their insights included recognizing connections between how they were raised to address conflict and how they currently address it. Comments included observations that positioned conflict along a continuum of being unproductive and to be avoided to potentially productive and to be encouraged. This raised some questions about the challenge of changing beliefs, attitudes and behaviors that have been in place for most of their lives. There was a hint of recognizing the challenge that some behaviors were based on assumptions that contributed to resistance to change [13].

Reflections included comments about how context impacted their choices of conflict styles. A predominant theme in this discussion was how levels of authority impacted approaches to conflict. Examples included participants noting that their approach to conflict in their classroom or research lab was different than their approach in interactions with their department heads. When asked about conflict with peers, the comments included a wide array of responses.

Because the TKI is identifying preferred conflict management styles, it is important to also look at the second most preferred style to get a sense of the range of styles a person may employ. The overall distribution of preferred styles in the general population suggests a level of flexibility and adaptability based on different situations. This is more noticeable when TKI scores are viewed as high, medium and low. Given this framework, participants were encouraged to look at their scores to also identify their second most preferred style. This was done to consider how likely they were to have and be able to apply a secondary approach. The results showed that 61% of the participants had at least one additional style that was within 20 percentage points of their preferred style.

### Peer support setting GROW goals. Follow up workshop

**Fig. 2:**
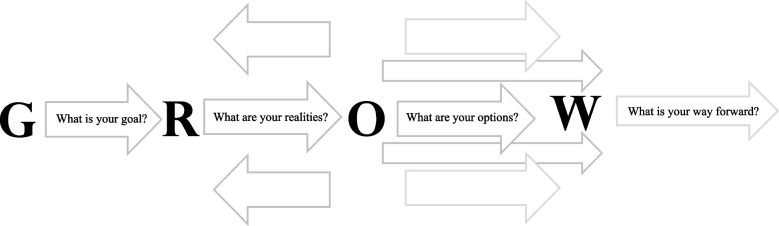
GROW goal setting model

In order to meet the session objectives of identifying strengths and development areas, and developing action plans to address them, participants were asked to prepare to share “why” they wanted to be a leader, to identify an upcoming opportunity to address conflict in an effective way, what strength they could bring to that interaction, and what development concern they needed to address. Participants were placed in pairs and provided the GROW coaching model (Fig. 2) outline to shape the conversation. The GROW Model [14] initially developed by Sir John Whitmore is a popular coaching model for problem solving and goal setting. It is used both for its simplicity of outlining steps and associated questions that help clarify goals and action items. It is also used for its contribution to uncovering perspectives and options that may not have been considered. In using a simplified GROW approach, the expectation was to share their Goal, the Realities of their situation, their idea of Options of how to meet the Goal, and their intended WAY Forward. The peer partner asked questions along the way to clarify information, to challenge assumptions, and to offer additional options to consider.

The Peer Coaching pair was also intended to establish commitments to follow up with each other regarding progress toward the goals. A follow-up workshop was offered for those who wanted additional coaching and support in understanding their “why” as a leader, and their range of options for managing conflict.

### Self-reflections

Participants were asked to share their insights from the workshop to offer what they considered of value, and to volunteer what they thought they would do differently because of the workshop. Several comments were offered confirming that the TKI data was what they expected based on their self-assessment or in accordance with what others have said about them. The comments related to value of the workshop included references to how helpful it was to see the range of conflict styles. This apparently aided several participants in understanding differences and how to manage interactions with different styles.

In response to what they would do differently, comments fell into three categories. Several participants mentioned their intentions to do more with crafting their “why” statements. Others stated they would be more conscious of opportunities to adjust their styles based on situations and styles of others. A third category of comments centered around planning to spend more time understanding what was driving their preference in a specific style.

## Discussion

Accelerating career transitions in academia benefit from intentionally articulating personal reasons for wanting to be in leadership roles defined behaviorally as influencing and impacting the way others feel, think, and behave. One critical dimension that contributes to being effective in leadership roles is the person’s effectiveness in managing conflict. The Thomas-Kilmann Conflict Styles Indicator, like other similar assessments, outlines different styles and suggests that having a range of styles enables a person to adapt to a variety of situations. Recognizing, understanding, and managing different approaches to conflict can be enhanced by focused training, skill practice, and application.

The participants in this ACT sample showed a preferred approach to a Compromising conflict style. Sixty one percent of the participants also show a strong secondary preferred style suggesting they have options in their conflict management styles. While being able to apply different conflict management styles depending on the situation is hypothesized as a targeted profile, participants recognized that some situations, and their own personal experiences, beliefs and attitudes regarding conflict may contribute to their resistance to switch from a preferred style to another one.

This workshop was presented to a small sample of participants. It was influenced by a larger sample of academic leaders in different contexts but all of the data is still drawn from qualitative input from training applications. Consequently, the hypotheses and conclusions generated here can benefit from more structured research and quantitative analyses. Variations in the observations that may have been influenced by various demographic characteristics were not explored. Though TKI reports no statistically significant differences based on race or gender, the phenomenological experiences reported by some participants about the risks or different conflict styles for women and Persons of Color is worth further exploration.

A final observation is a recognition that this workshop was primarily focused on increasing knowledge regarding conflict styles. It included a focus on setting measurable GROW goals to apply that knowledge. Soliciting feedback from participants about outcomes of those goals and their insights about the application of specific conflict styles is recommended as a follow-up. Additional research might also focus on the primary assumption about the importance of managing conflict to career movement in academic leadership and effectiveness in that role.

## Conclusions

A basic understanding of cell biology provides an effective framework for understanding leadership, conflict, and change management. This small sample of underrepresented academic leaders in STEM demonstrated a preference for a Compromising conflict management style. This may contribute to avoiding constructive conflict as a leader by using a Collaborative conflict management style. The use of focused goals to leverage strengths and address developmental opportunities served as one step to applying individual feedback to changes in behavior. Follow-up on those goals would provide more insight. The sample size and data analysis suggest hypotheses that may be addressed with more robust research. This sample size provides more information about individual approaches to conflict than it does about a population of under-represented academic leaders in STEM.

## Data Availability

The datasets used and/or analyzed during the current study are available from the corresponding author on reasonable request.

## Abbreviations

ACT: Accomplishing Career Transitions
ASCB: American Society of Cell Biologists
GROW: Goal, Realities, Options, Way Forward
TKI: Thomas Kilmann Conflict Styles Indicator
